# Antimicrobial Susceptibility of* Escherichia coli* Strains Isolated from* Alouatta* spp. Feces to Essential Oils

**DOI:** 10.1155/2016/1643762

**Published:** 2016-05-30

**Authors:** Valéria Maria Lara, Adriano Bonfim Carregaro, Deise Flores Santurio, Mariangela Facco de Sá, Janio Moraes Santurio, Sydney Hartz Alves

**Affiliations:** ^1^Department of Veterinary Medicine, University of São Paulo, Avenida Duque de Caxias Norte 225, 13635-900 Pirassununga, SP, Brazil; ^2^Department of Microbiology and Parasitology, Federal University of Santa Maria, Avenida Roraima 1000, 97105-900 Santa Maria, RS, Brazil

## Abstract

This study evaluated the* in vitro *antibacterial activity of essential oils from* Lippia graveolens* (Mexican oregano),* Origanum vulgaris *(oregano),* Thymus vulgaris *(thyme),* Rosmarinus officinalis *(rosemary),* Cymbopogon nardus *(citronella),* Cymbopogon citratus *(lemongrass), and* Eucalyptus citriodora* (eucalyptus) against* Escherichia coli *(*n* = 22) strains isolated from* Alouatta *spp. feces. Minimum inhibitory concentration (MIC) and minimum bactericidal concentration (MBC) were determined for each isolate using the broth microdilution technique. Essential oils of Mexican oregano (MIC mean = 1818 *μ*g mL^−1^; MBC mean = 2618 *μ*g mL^−1^), thyme (MIC mean = 2618 *μ*g mL^−1^; MBC mean = 2909 *μ*g mL^−1^), and oregano (MIC mean = 3418 *μ*g mL^−1^; MBC mean = 4800 *μ*g mL^−1^) showed the best antibacterial activity, while essential oils of eucalyptus, rosemary, citronella, and lemongrass displayed no antibacterial activity at concentrations greater than or equal to 6400 *μ*g mL^−1^. Our results confirm the antimicrobial potential of some essential oils, which deserve further research.

## 1. Introduction

The indiscriminate use of antibacterial agents has led to one of the largest recent global health problems which is the emergence of bacterial resistance. Several bacteria genera have developed multidrug resistance, including* Escherichia coli *[1].* E. coli *are Gram-negative, nonsporulating facultative anaerobes, found primarily in the gastrointestinal tract of different species of domestic and wild animals and environments such as soil, water, and plants [2]. This pathogen can cause mild to severe infection, possibly leading to death from septicemia depending on the bacterial strain and its virulence, as well as host-related factors such as age and immunity [3].

In nonhuman primates, enteric infection by* E. coli* and the isolation of pathogenic strains from healthy animals have been documented [4]. Moreover, there are reports of the isolation of resistant and multidrug-resistant* E. coli* strains from wild animals [5–7]. This finding is important given the increased contact between wild animals and humans, enabling cross-species transmission (CRT) of these bacteria. Additionally, the synthesis of new antimicrobials has declined in recent years. As such, new treatment options are needed to overcome bacterial resistance.

Essential oils (EOs) are volatile and complex natural products derived from the secondary metabolism of plants and can be found in different plant parts, including the leaves and stalk. EOs probably consist of 20 to 60 different compounds, in which at least two or three are in higher concentrations, depending on the EOs [8]. These compounds exhibit significant therapeutic and pharmacological potential as well as antimicrobial properties, already established for Gram-positive bacteria and Gram-negative bacteria found in different animals species, including humans [7–16]. The main compounds with possible antimicrobial activity are terpinenes, cymenes, thymol, and carvacrol [17–20]. This antimicrobial effect is mainly related to changes on bacterial cell membrane permeability and integrity [21].

The Howler monkey is a primate from the family Atelidae and genus* Alouatta. *It is widely distributed, occurring from the states of Bahia to Rio Grande do Sul, and is listed as an endangered species [22]. Howlers are an arboreal species and their diet consists mainly of fruit, leaves, seeds, and flowers [23]. In Brazil, it is common practice to keep monkeys and other wild animals in captivity, where they are generally treated like humans. The animals are fed and medicated indiscriminately, largely receiving antibacterial agents. In addition, direct owner-animal contact is a public health problem, given the possible transmission of infectious zoonotic microorganisms as well as drug-resistant bacteria.

The aim of our study was to evaluate the susceptibility of 22* Escherichia coli *strains isolated from captive Howler monkeys (*Alouatta*) to seven essential oils, in order to assess their potential use as an alternative treatment for* E. coli *infection.

## 2. Materials and Methods

### 2.1. *Escherichia coli* Strains Tested

We studied 22 strains of* E. coli, *isolated from the feces of Howler monkeys (*Alouatta *spp.) with diarrhea from the Anaerobe Laboratory of Universidade Federal de Santa Maria (UFSM).

### 2.2. Essential Oils

The essential oils tested were Mexican oregano (*Lippia graveolens*), oregano (*Origanum vulgare*), rosemary (*Rosmarinus officinalis*), eucalyptus (*Eucalyptus citriodora*), citronella (*Cymbopogon nardus*), lemongrass (*Cymbopogon citratus*), and thyme (*Thymus vulgaris*). Mexican oregano essential oil was purchased from Agroindustrial Don Pablo (Chihuahua, CHIH, Mexico). Oregano, rosemary, eucalyptus, citronella, lemongrass, and thyme essential oils were purchased from Essential 7 (Roswell, New Mexico, USA), and all of them came in sealed amber glass bottles. EOs selection was based on previous studies from our laboratory and other studies [10–12, 19, 24, 25].

### 2.3. Minimum Inhibitory Concentration (MIC)

The essential oils were weighed (1 g), diluted in methanol to a concentration of 640 mg mL^−1^ (solution I), and then diluted in Müller-Hinton broth at a proportion of 1 : 100, obtaining a concentration of 6400 *μ*g mL^−1^ (solution II). Based on standard M7-A7 of the Clinical and Laboratory Standards Institute (CLSI) (2006) [26], 100 *μ*L volumes of Müller-Hinton broth were distributed on a microtiter plate. Next, serial dilution was performed with solution II, obtaining final concentrations of 3200, 1600, 800, 400, 200, and 100 *μ*g mL^−1^. The* E. coli* were cultivated in Müller-Hinton agar and the colonies were then suspended in 0.085% saline solution, producing turbidity equivalent to McFarland Standard number 0.5 (1 × 10^8^ UFC mL^−1^). Each well containing essential oils was then inoculated with 10 *μ*L (1 × 10^5^ UFC mL^−1^) of this suspension. The microplates were incubated aerobically at 35°C/24 h. The MIC is the lowest concentration of essential oil that will inhibit bacterial growth. Positive controls for inocula growth as well as solvent and negative (medium alone) controls were included. All experiments were performed in triplicate.

### 2.4. Minimum Bactericidal Concentration (MBC)

Minimum bactericidal concentration is the lowest concentration of essential oils required to kill the inoculum and was determined by the wells with no visible bacterial growth after 24 h of incubation. A 10 *μ*L aliquot was transferred from these wells to the surface of the Müller-Hinton agar. Essential oil concentration declined after 24 h incubation at 35°C, with no bacterial growth observed. Experiments were performed in triplicate.

## 3. Results and Discussion

In recent years, research has been conducted on the susceptibility of essential oils and their chemical compounds to different bacteria species isolated from domestic animals, humans, and food [9, 11, 12, 20, 27, 28]. The present study is the first to present results on the susceptibility of 22* E. coli *strains from Howler monkeys (*Alouatta *spp.) to seven essential oils.

The oil from* Lippia graveolens *was the most effective essential oil against the 22* E. coli *strains tested, of which 27.2% were inhibited at a concentration of 800 *μ*g mL^−1^ and 100% were inhibited by a concentration of 3200 *μ*g mL^−1^ (mean MIC = 1818 *μ*g mL^−1^; mean MBC = 2618 *μ*g mL^−1^) (Table 1). This study found similar results to those reported in a previous investigation showing a moderate antimicrobial effect of Mexican oregano against* E. coli *isolated from poultry and cattle [12]. However, based on the MIC and MBC values obtained here, it can be inferred that the* E. coli* strains isolated from Howler monkeys showed greater susceptibility to essential oil of Mexican oregano than those isolated from poultry and cattle [12].

Results obtained for thyme (*Thymus vulgaris*) indicated a lower bactericidal effect and susceptibility than* L. graveolens*, with only 36.36% (8/22) of the 22* E. coli *strains tested inhibited by a concentration of 600 *μ*g mL^−1^ and 63.63% (14/22) inhibited at 3200 *μ*g mL^−1^ (mean MIC = 2618 *μ*g mL^−1^; mean MBC = 2909 *μ*g mL^−1^) (Table 1). Other studies also show the antibacterial activity of* T. vulgaris *against* E. coli *strains isolated from different animal species [12, 18, 20]. However, Sartoratto et al. (2004) [18] found no effect for* T. vulgaris *against* E. coli *CCT0547.

The major compounds of* L. graveolens *and* T. vulgaris* EOs are *o*-cymene, *γ*-terpinene, thymol, and carvacrol [17, 19]. These compounds have shown antimicrobial activity against some bacteria, especially* E. coli* strains [8, 10, 17, 27]. Although the chemical compounds from the EOs used in our study were not analyzed, it could be suggested that *o*-cymene, *γ*-terpinene, thymol, and carvacrol were responsible for the antimicrobial effect against* E. coli* strains isolated from Howler monkeys, since those EOs were already evaluated in a previous study of our group [19]. However, further studies evaluating these constituents separately are necessary to assess their individual activity against* E. coli* strains.

Oregano (*Origanum vulgare*) is widely used as seasoning in several countries and its antimicrobial activity has also been demonstrated. In our experiment, essential oil of oregano showed lower antimicrobial activity than that of* L. graveolens *and* T. vulgaris* against 100% of the* E. coli *used (mean MIC = 3418 *μ*g mL^−1^; mean MBC = 4800 *μ*g mL^−1^) (Table 1). MIC values (between 1600 and 3200 *μ*g mL^−1^) were lower than those recorded against* E. coli *from poultry and cattle [12]. However, our findings differed significantly from those reported in another study [18], where the authors tested an* E. coli *standard strain (CCT0547) and found no antimicrobial effect. By contrast,* Salmonella enterica* strains from poultry were highly susceptible to antibacterial treatment with oregano when compared to thyme [11]. It is important to underscore that the main components of oregano essential oil are carvacrol (at 66%–92.6% concentration), cymene (4.6%–9.2%), and thymol (1.0%–1.9%) [19, 20], suggesting that* E. coli *strains from Howler monkeys may be more susceptible to thymol than carvacrol or the thymol and carvacrol combination. However, further research is needed to confirm this hypothesis.

The other four essential oils tested, namely, rosemary (*Rosmarinus officinalis*), eucalyptus (*Eucalyptus citriodora*), citronella (*Cymbopogon nardus*), and lemongrass (*Cymbopogon citratus*), showed no antibacterial effect against the 22* E. coli *strains studied. Our results differed from those found by other researchers, who observed antimicrobial activity in these oils against Gram-positive and Gram-negative bacteria [10, 13, 14, 16]. Studies indicate that how essential oils are obtained, the season, and geographic distribution are factors that can change the composition of these oils and alter their antimicrobial properties [28, 29], thus explaining the different results obtained in a number of studies on plant essential oils.

In conclusion, the essential oils of* Lippia graveolens*,* Thymus vulgaris*, and* Origanum vulgare *used in the present study show potential for use as antibacterial agents against* E. coli* strains. Moreover, based on our findings, it can be assumed that *o*-cymene, *γ*-terpinene, thymol, and carvacrol were the active ingredients with the highest antimicrobial effect* in vitro* against* E. coli *strains.

## Figures and Tables

**Table 1:** MIC and MBC of seven essential oils.

Essential oils	MIC (*μ*g mL^−1^)				MBC (*μ*g mL^−1^)			
	Band	MIC_50_	MIC_90_	Mean	Band	MBC_50_	MBC_90_	Mean
*Lippia graveolens*	800–3200	1600	3200	1818	800–3200	3200	3200	2618
*Thymus vulgaris*	1600–3200	3200	3200	2618	1600–3200	3200	3200	2909
*Origanum vulgare*	1600–3200	3200	3200	3418	3200–6400	3200	6400	4800
*Rosmarinus officinalis*	>6400	>6400	>6400	ND	0	0	0	ND
*Eucalyptus citriodora*	>6400	>6400	>6400	ND	0	0	0	ND
*Cymbopogon nardus*	>6400	>6400	>6400	ND	0	0	0	ND
*Cymbopogon citratus*	>6400	>6400	>6400	ND	0	0	0	ND

MIC: minimum inhibitory concentration; MBC: minimum bactericidal concentration; ND: not determined.

## References

[1] Högberg L. D., Heddini A., Cars O. (2010). The global need for effective antibiotics: challenges and recent advances. *Trends in Pharmacological Sciences*.

[2] Manges A. R., Johnson J. R. (2005). Reservoirs of extraintestinal pathogenic *Escherichia coli*. *Microbiology Spectrum*.

[3] Beutin L. (1999). *Escherichia coli* as a pathogen in dogs and cats. *Veterinary Research*.

[4] Carvalho V. M., Gyles C. L., Ziebell K. (2003). Characterization of monkey enteropathogenic *Escherichia coli* (EPEC) and human typical and atypical EPEC serotype isolates from neotropical nonhuman primates. *Journal of Clinical Microbiology*.

[5] Jobbins S. E., Alexander K. A. (2015). From whence they came—antibiotic-resistant *Escherichia coli* in African wildlife. *Journal of Wildlife Diseases*.

[6] Marinho C., Igrejas G., Gonçalves A. (2014). Azorean wild rabbits as reservoirs of antimicrobial resistant *Escherichia coli*. *Anaerobe*.

[7] Silva N., Igrejas G., Figueiredo N. (2010). Molecular characterization of antimicrobial resistance in enterococci and Escherichia coli isolates from European wild rabbit (*Oryctolagus cuniculus*). *Science of the Total Environment*.

[8] Thapa D., Losa R., Zweifel B., Wallace R. J. (2012). Sensitivity of pathogenic and commensal bacteria from the human colon to essential oils. *Microbiology*.

[9] Hernández T., Canales M., Avila J. G. (2003). Ethnobotany and antibacterial activity of some plants used in traditional medicine of Zapotitlán de las Salinas, Puebla (México). *Journal of Ethnopharmacology*.

[10] Millezi A. F., Baptista N. N., Caixeta D. S., Rossoni D. F., Cardoso M. G., Piccoli R. H. (2014). Chemical characterization and antibacterial activity of essential oils from medicinal and condiment plants against *Staphylococcus aureus* and *Escherichia coli*. *Revista Brasileira de Plantas Medicinais*.

[11] Santurio J. M., Santurio D. F., Pozzatti P., Moraes C., Franchin P. R., Alves S. H. (2007). Antimicrobial activity of essential oils from oregano, thyme and cinnamon against Salmonella enterica sorovars from avian source. *Ciencia Rural*.

[12] Santurio D. F., da Costa M. M., Maboni G. (2011). Antimicrobial activity of spice essential oils against *Escherichia coli* strains isolated from poultry and cattle. *Ciencia Rural*.

[13] Scherer R., Wagner R., Duarte M. C. T., Godoy H. T. (2009). Composition and antioxidant and antimicrobial activities of clove, citronella and palmarosa essential oils. *Revista Brasileira de Plantas Medicinais*.

[14] Silva M. T. N., Ushimaru P. I., Barbosa L. N., Cunha M. L. R. S., Fernandes A. (2009). Antibacterial activity of plant essential oils against *Staphylococcus aureus* and *Escherichia coli* strains isolated from human specimens. *Revista Brasileira de Plantas Medicinais*.

[15] Thosar N., Basak S., Bahadure R. N., Rajurkar M. (2013). Antimicrobial efficacy of five essential oils against oral pathogens: An in vitro study. *European Journal of Dentistry*.

[16] Zago J. A. A., Ushimaru P. I., Barbosa L. N., Fernandes A. (2009). Synergism between essential oils and antimicrobial drugs against *Staphylooccus aureus* and *Escherichia coli* strains from human infections. *Brazilian Journal of Pharmacognosy*.

[17] Marino M., Bersani C., Comi G. (1999). Antimicrobial activity of the essential oils of *Thymus vulgaris* L. measured using a bioimpedometric method. *Journal of Food Protection*.

[18] Sartoratto A., Machado A. L. M., Delarmelina C., Figueira G. M., Duarte M. C. T., Rehder V. L. G. (2004). Composition and antimicrobial activity of essential oils from aromatic plants used in Brazil. *Brazilian Journal of Microbiology*.

[19] Pozzatti P., Scheid L. A., Spader T. B., Atayde M. L., Santurio J. M., Alves S. H. (2008). In vitro activity of essential oils extracted from plants used as spices against fluconazole-resistant and fluconazole-susceptible *Candida* spp.. *Canadian Journal of Microbiology*.

[20] Höferl M., Buchbauer G., Jirovetz L. (2009). Correlation of antimicrobial activities of various essential oils and their main aromatic volatile constituents. *Journal of Essential Oil Research*.

[21] Lambert R. J. W., Skandamis P. N., Coote P. J., Nychas G.-J. E. (2001). A study of the minimum inhibitory concentration and mode of action of oregano essential oil, thymol and carvacrol. *Journal of Applied Microbiology*.

[22] Instituto Chico Mendes de Conservação da Biodiversidade (ICMBio) Lista Oficial da Fauna Brasileira ameaçada de extinção. http://www.icmbio.gov.br/portal/biodiversidade/fauna-brasileira/lista-de-especies.html.

[23] Silva R. M., Marques J. C. B., Ferreira H. D. Dados preliminares sobre a dieta do Bugio *Alouatta caraya* (Primates, Cebidae) na área do Parque Zoológico de Goiânia-PZG.

[24] Chaibi A., Ababouch L. H., Belasri K., Boucetta S., Busta F. F. (1997). Inhibition of germination and vegetative growth of *Bacillus cereus* T and *Clostridium botulinum* 62A spores by essential oils. *Food Microbiology*.

[25] Santos Pereira R., Sumita T. C., Furlan M. R., Cardoso Jorge A. O., Ueno M. (2004). Antibacterial activity of essential oils on microorganisms isolated from urinary tract infection. *Revista de Saúde Pública*.

[26] Clinical and Laboratory Standards (2006). Methods for dilution antimicrobial susceptibility tests for bacteria that grow aerobically. *Approved Standard*.

[27] Arana-Sánchez A., Estarrón-Espinosa M., Obledo-Vázquez E. N., Padilla-Camberos E., Silva-Vázquez R., Lugo-Cervantes E. (2010). Antimicrobial and antioxidant activities of Mexican oregano essential oils (*Lippia graveolens* H. B. K.) with different composition when microencapsulated in *β*-cyclodextrin. *Letters in Applied Microbiology*.

[28] Marco C. A., Innecco R., Mattos S. H., Borges N. S., Nagao E. O. (2007). Características do óleo essencial de capim-citronela em função de espaçamento, altura e época de corte. *Horticultura Brasileira*.

[29] Blank A. F., Costa A. G., Arrigoni-Blank M. D. F. (2007). Influence of season, harvest time and drying on Java citronella (*Cymbopogon winterianus* Jowitt) volatile oil. *Brazilian Journal of Pharmacognosy*.

